# Visual Echolocation Concept for the Colorophone Sensory Substitution Device Using Virtual Reality

**DOI:** 10.3390/s21010237

**Published:** 2021-01-01

**Authors:** Patrycja Bizoń-Angov, Dominik Osiński, Michał Wierzchoń, Jarosław Konieczny

**Affiliations:** 1 Consciousness Lab, Institute of Psychology, Jagiellonian University, 30-060 Kraków, Poland; michal.wierzchon@uj.edu.pl; 2 Department of Electronic Systems, Norwegian University of Science and Technology, NO-7491 Trondheim, Norway; dominik.osinski@ntnu.no; 3 Jagiellonian Human-Centered Artificial Intelligence Laboratory, Jagiellonian University, 30-348 Kraków, Poland; 4 Department of Process Control, AGH University of Science and Technology, 30-059 Kraków, Poland; koniejar@agh.edu.pl

**Keywords:** 3D camera, stereo vision, auditory SSD, distance sonification, colour sonification, 3D scene sonification, virtual reality

## Abstract

Detecting characteristics of 3D scenes is considered one of the biggest challenges for visually impaired people. This ability is nonetheless crucial for orientation and navigation in the natural environment. Although there are several Electronic Travel Aids aiming at enhancing orientation and mobility for the blind, only a few of them combine passing both 2D and 3D information, including colour. Moreover, existing devices either focus on a small part of an image or allow interpretation of a mere few points in the field of view. Here, we propose a concept of visual echolocation with integrated colour sonification as an extension of Colorophone—an assistive device for visually impaired people. The concept aims at mimicking the process of echolocation and thus provides 2D, 3D and additionally colour information of the whole scene. Even though the final implementation will be realised by a 3D camera, it is first simulated, as a proof of concept, by using VIRCO—a Virtual Reality training and evaluation system for Colorophone. The first experiments showed that it is possible to sonify colour and distance of the whole scene, which opens up a possibility to implement the developed algorithm on a hardware-based stereo camera platform. An introductory user evaluation of the system has been conducted in order to assess the effectiveness of the proposed solution for perceiving distance, position and colour of the objects placed in Virtual Reality.

## 1. Introduction

Perception of shape, colour and depth are basic functions of the visual system that contribute to orientation and mobility in the surrounding environment. Therefore, many Sensory Substitution Devices (SSDs) are designed with the aim of coding shape [1,2], colour [3,4] and distance [3,5,6,7] in the form of non-visual stimuli, in order to provide the information for those who cannot access them through vision. When thinking about the navigation and mobility of the visually impaired, some of the information, especially distance, is crucial. Being aware of the close environment, finding objects, obstacles or paths is what makes their mobility easier or even possible. There are several ways to obtain and pass such information. Some devices [2,4] scan a captured 2D scene image from left to right informing about the position of objects, their colour or light intensity in an audio form. Other SSDs e.g., [5,6] translate distance information into audio and/or haptic output providing constant feedback to help to navigate. Finally, there are solutions providing both distance and colour information e.g., [7,8,9] but with some limitations. Often, the colour data are simplified into black and white or light intensity information. To the authors’ knowledge, there are only three devices [3,7,10] that enable distance and colour perception simultaneously. However, the first one provides such information only for a single point chosen from the image, the second one from several image points, whereas, in the third system, feedback is presented in a text-to-speech form. Thus, even though various solutions have been presented throughout the years, all of them provide only a limited amount of data, without combining 2D, 3D and colour information of the whole scene.

On the other hand, it is also known that some visually impaired people are able to use echolocation to assess the position, size, distance, shape and materials of the perceived objects [11]; however, the upper limit of the perceivable sound frequencies determines the size of the objects that can be registered in this manner. Some earlier works suggest that it is possible to develop an effective system where the generated ultrasonic signals are sent out and then the ultrasonic echoes are stretched in time in order to make them perceivable by humans [12,13]. Inspired by these findings, the authors decided to develop a sensory substitution system that combines some features of echolocation with the colour sonification method used in an existing SSD: Colorophone [3,14]. The proposed functionality aims to mimic echolocation and allows users to perceive the surrounding environment more completely, by informing about colour as well as 2D/3D features of the whole scene.

Colorophone is a wearable, real-time sensory substitution system that provides colour and distance information. It utilises an RGB camera and an ultrasonic distance sensor, providing simultaneous sonification of data from both. For the camera, the region of interest is the centre of the image, from where the information about the colour is acquired and averaged, while the ultrasonic sensor enables measurement of the distance to the closest object in the region of interest. The obvious limitation of the system is the ability to represent colour and distance information only from a narrow area. In order to extend the colour and distance sonification functionality to the whole visual scene acquired by the camera, authors proposed a prototype that demonstrates the proof of concept of the next generation of Colorophone, which is based on stereo vision and utilises vision-based “echolocation”. The novelty of this solution is that it combines colour sonification with an integrated depth measurement of the whole field of view.

Advances in 3D camera systems development and exponential growth in processing capability of computational units open new possibilities for sensory substitution solutions development. Nowadays, cameras provide high-resolution images with excellent colour reproduction at a high-frame rate. When thinking about Colorophone’s development, using these new opportunities can solve the problem of sensors integration. This is because, from a stereo camera, we can acquire information about colour and depth that covers a substantial part of the surroundings. At this stage of the prototype development, we aim at simulating the 3D camera usage in virtual reality, in order to evaluate the suitability of the proposed solution for the natural scene perception. Some of the advantages of that solution are the elimination of additional costs and full control over the camera system and the scene parameters. For this purpose, we utilise VIRCO—a virtual reality training and evaluation system for Colorophone SSD [15].

Another challenge addressed in this paper is the selection of how the information is presented to the user. Human vision typically acquires much more data than a sense of hearing can represent. Therefore, it is not obvious how to solve this problem in visual to auditory SSDs. This is why the authors decided to mimic echolocation systems existing in nature. Echolocation is the ability to perceive the physical environment by using reflected sound waves. Humans can use echolocation to some extent, and particularly visually impaired people are capable of benefitting from it [16]. They make use of self-emitted sounds, like ones generated by voice, or those generated by mechanical means, such as shoes, cane or specially designed devices emitting clicks. Through echolocation, the blind are capable of distance discrimination [17], object localisation [18,19] and obstacle detection [20] with considerable precision that brings great potential when thinking about the independent orientation and mobility of blind people. The visual echolocation concept aims at combining features of echolocation and the Colorophone sensory substitution system by utilising stereo vision. One can imagine the proposed solution as an axial plane facing a user that moves away through objects in a scene. Whatever the axial plane crosses at a given moment, it is being sonified. The concept, as well as its visualisation, is presented in link 1 [21].

## 2. Related Work

Various SSDs are aimed at delivering information useful for navigation, orientation, objects finding, text recognition, face perception, etc. They utilise different approaches and provide various types of feedback. For example, EyeMusic introduced by Abboud et al. [4] is based on a camera mounted on glasses and bone-conductive headphones. Images captured by the camera are sonified from left to right, assigning high pitch sounds to objects that are higher, and low pitch sounds to objects that are lower in the scene. Sound amplitude corresponds to brightness so that brighter elements are louder. Additionally, different musical instruments represent five basic colours. Caraiman et al. [5] presented SoundOfVision—a stereo vision solution enabling obtaining a depth map and informing users about the distance to objects in an audio and haptic form. Distance is coded by the intensity of the feedback. Moreover, the device also enables text recognition. Colour information is used to improve the depth map precision but is not sonified. Another stereo vision-based approach has been described by Bujacz et al. [6]. Users receive information about distance via spatialised musical sounds (or vibrations). The distance is mapped onto loudness, frequency and temporal delay. Similarly to SoundOfVision [5], there is no colour sonification. Hamilton-Fletcher et al. [22] introduced a novel SSD implemented on an iPhone. It uses the phone’s inbuilt single or dual-lens camera, an external infrared depth sensor, a thermal sensor or stored images and movies; however, only one source of the signal may be used at a time. Users can select the part of the scene that is to be sonified. Close objects are heard as loud, while far as quiet. Only black or white colour is represented. Another device described by González-Mora et al. [9] consists of two cameras capturing a depth map that is further converted into auditory “clicks”, one for each of the positions previously registered in the depth map. All sounds are auralised—when perceived through headphones, they seem to approach from a certain position in the environment. The device is able to encode up to 16 levels of grayscale.

As mentioned above, there are only two SSDs besides Colorophone that enable simultaneous colour and distance sonification. The first has been presented by Hub et al. [10]—the prototype consists of a sensor module with a detachable cane and a portable computer. The sensor module is equipped with two cameras, a keyboard, a digital compass, a 3D inclinometer, and a loudspeaker. It can be handled like a flashlight. Different sequences and loudness options can be chosen with designated keys. Various inquiries concerning an object’s features can be sent to the portable computer. These inquiries are answered via a text-to-speech engine. Users can obtain information such as colour of the front-most object, its estimated size, and distance to the current user position. Moreover, each object in the scene can contain additional information, like warnings or detailed object descriptions that can then be passed to the blind user.

The second SSD coding both distance and colour was presented by Gomez et al. [7]. It consists of a 3D camera, bone-conduction headphones, a laptop and optionally a tablet. Colour information is coded by instruments and distance by the duration of the sound (the closer the shorter). In local mode, information is provided from a central row of 25 points coded from left to right. In global mode, users can scan the image by sliding finger(s) on the tablet, and as many points as the number of involved fingers can be accessed simultaneously. To sum up, most of the previously proposed SSDs do not sonify both 2D/3D and colour information, and those capable to do so are still limited.

## 3. Extension Tool’s Simulation for Colorophone SSD

The algorithm realising the visual echolocation concept presented in this paper can be explained in three steps: the way scenes are captured, methods used for processing the captured images, and the sonification approach. The first step needs to be simulated and is the only one that differs from a procedure when using a setup with a real stereo camera system as compared to the VR simulation. The two latter ones can be applied as well in a system with a 3D camera. LabVIEW from National Instruments (NI) [23] was chosen as a working environment, as it is convenient for prototyping and additionally it includes a Vision Module to work with stereo vision.

### 3.1. 3D Camera Simulation Using VIRCO

VIRCO [15] is a Virtual Reality based tool for usability training of the Colorophone device. It was designed to make the learning process of the Colorophone sonification method easier both for the user and the researcher. VIRCO eliminates the influence of environmental factors that can disturb the usage of the device and the acquisition of its functional principles. This tool is especially useful in the process of the development of a new system. This is because, when using a real camera, one will never get an ideal scene representation, which helps to test the concept of visual echolocation. In particular, in real-life conditions, the acquired depth map is based on approximation rather than an exact reflection of the true scene. Particularly, VIRCO application eliminates problems caused e.g., by variable light conditions. Importantly, it also provides a cost-effective approach to verify whether the concept of visual echolocation should be further developed. Not only can a camera be simulated, but also its various parameters that would otherwise require buying different cameras in order to choose the best fit. Moreover, the approach gives a possibility to test different settings of sonification, such as various sounds, length of the sounds, etc.

In order to construct visual scenes, the working environment of VIRCO—Unity [24] was used. Two virtual cameras were implemented with a defined position and baseline (distance between each other) in order to simulate a stereo vision system. Additional parameters, such as field of view (horizontal and vertical), resolution and aspect ratio of each camera, were also set. The system described above allows saving images from the virtual cameras separately.

### 3.2. Depth Map Acquisition

**Figure 1:**
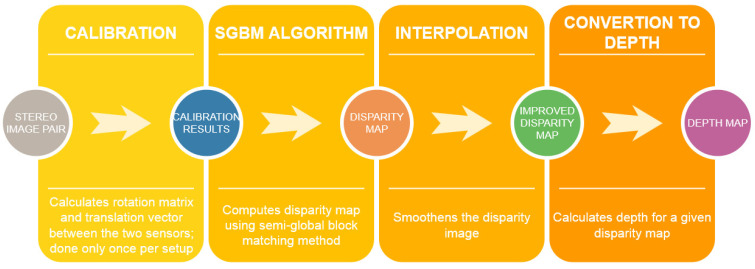
The procedure of computing depth map.

Regardless of the way scenes are captured—using the simulation in VIRCO or with a real 3D camera—from this point, the procedure is the same for both of them. Depth map acquisition is a process consisting of several steps (Figure 1).

Depth calculation is based on triangulation—a process of determining a point in 3D space given its projections onto two, or more images. However, the triangulation requires the parameters of the stereo system that is used, meaning camera matrices. These are calculated in a calibration step. So-called spatial calibration provides pixel to real-world unit transformations while accounting for many errors inherent to the imaging setup. The requirement is that the two cameras are parallel in a horizontal arrangement. Since the simulated setup is fully controllable, those conditions can be easily met. NI provides a calibration grid that needs to be defined in terms of a number of markers and distances between them before running the calibration. For the prototype testing, a grid implemented in Unity is utilised. During the calibration procedure, the grid has to be presented in front of the cameras while tilted at different angles. Based on grid dimensions, the rotation matrix and translation vector between the two cameras are computed as well as the essential and fundamental matrices. Moreover, each camera is additionally calibrated by calculating internal and external parameters. Calibration has to be done only once for a given setup and resolution, and the results can be saved in a file [23].

The next step in the procedure is a disparity map computation. The disparity is the planar difference between corresponding points in a reference image and a target image (i.e., images of the same scene captured by two cameras). The Semi Global Block Matching (SGBM) algorithm was chosen in order to calculate disparity. This algorithm is widely used in real-time stereo vision applications, such as robotics or advanced driver assistance systems [25]. The SGBM algorithm is pre-implemented in LabVIEW with optional parameter adjustments, such as correspondence or postfilter options. Moreover, the interpolation step smoothens the disparity image by approximating pixel values in case the SGBM algorithm cannot determine a disparity. The output of the step is an improved disparity map that can be further used to compute the depth map.

Finally, depth is calculated for each previously computed disparities. In LabVIEW, this is also realised by a built-in function. At this stage, as well as after computing the disparity map, it is already known whether the results are satisfactory, and, if not,-the parameters can be adjusted accordingly.

### 3.3. Visual Echolocation

**Figure 2:**
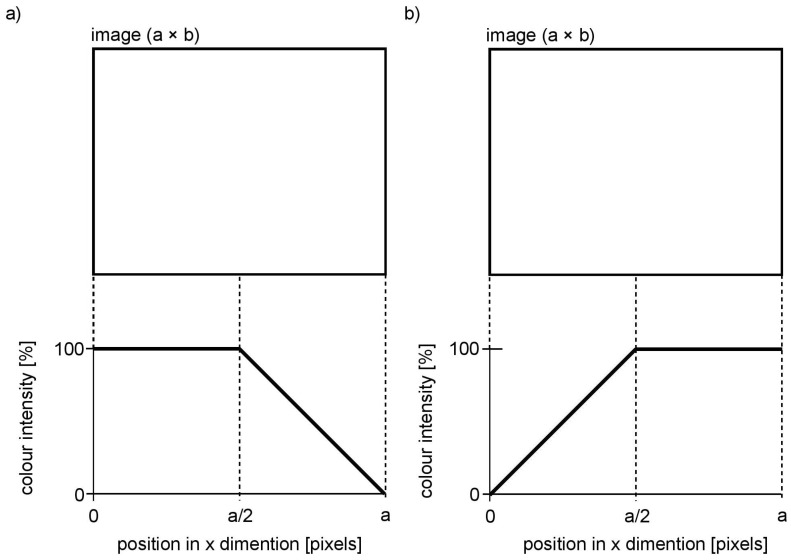
Linear functions applied to colour intensity to realise spatialised sound. (**a**) Left channel; (**b**) Right channel.

When the depth map is computed, the last step is to sonify the depth and colour information. Visual echolocation concept presented in this paper utilises the colour sonification method of Colorophone. Each of the registered colour components is represented by a specific sound compound of particular frequencies depending on RGB components, where blue is represented by the lowest frequency (250 Hz), green by the middle frequency (500 Hz), and red is represented by the highest frequency of the generated sound (1000 Hz). Additionally, the white colour component, calculated as the minimum value of all RGB components, which is then subtracted from all RGB values, is represented by white noise (i.e., black is associated with no sound). The loudness of the four main sound components (referring to the R, G, B, and white components) is proportional to the intensity of the given colour component. In order to represent colour and distance information about the whole scene at the same time, the concept of visual echolocation was implemented. More precisely, the depth map is split into axial planes of an adjustable thickness (Plane Averaging parameter, PA): for example, for a value of PA = 10 cm and depth limit of 500 cm, there will be 50 axial planes, first starting at a distance of 0 cm, second at 10 cm, and so on up to 500 cm. Each axial plane is sonified separately starting from the closest one and going smoothly through all the planes. The resulting sound represents the mean colour of all the pixels in a given plane as well as the size of a surface: the more intense the colour and the bigger the surface (i.e., it covers more pixels), the louder the sound. It is also possible to define the distance limits at which the scene sonification process starts and finishes. In the experiments described below the starting point for the moving sonification, the plane was set to 15 cm and the farthest sonified plane was chosen to be 500 cm in order to provide the information about the close environment. To give a user a sense of space, the sound is spatialised—it can be perceived as coming either from the left side, front or from the right side. This was realised by applying a linear function to the colour intensities, depending on the canal (Figure 2). The implemented spatialisation function is the first, basic attempt to introduce panning and does not include the interaural time difference principle. More sophisticated sound spatialisation will be implemented in the subsequent attempt to realise visual echolocation. For example, for the left channel (Figure 2a) all the pixels from the left to the middle of the image are sonified with 100% colour intensity, while pixels in the right half of the image are sonified with the colour intensity decreasing linearly from the middle to the right of the image. For the right channel, the process is mirrored respectively (Figure 2b).

## 4. Results

Several virtual models were designed in Unity to mimic real-life conditions under which the tool might be useful. Table 1 presents the parameters used for the first tests.

In the simulation, the resolution of images can be freely selected. However, for a possible future application of the prototype with a real 3D camera, a resolution of 1104 × 621 was chosen for the conversion to the depth maps. Higher and lower resolutions were also considered; nevertheless, the aforementioned one provides a compromise between processing time and quality of the computed depth map (Table 2). For the following steps of the conversion, the resolution might be decreased in order to reduce the computation time. Table 2 presents execution times required for a given depth map calculation as well as for the execution of the entire procedure of visual echolocation, including displaying the visual and audio results for different image resolutions. The resolution for further data processing was chosen at 368 × 207. The higher one would cause too long of a processing time, while, with the lower one, the results are still acceptable, but fewer details can be distinguished.

**Figure 3:**
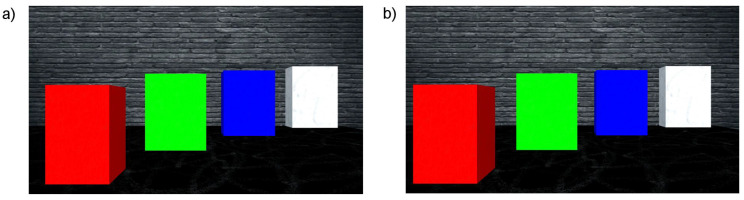
Example of a simulated scene. (**a**) View from left camera; (**b**) View from right camera.

**Figure 4:**
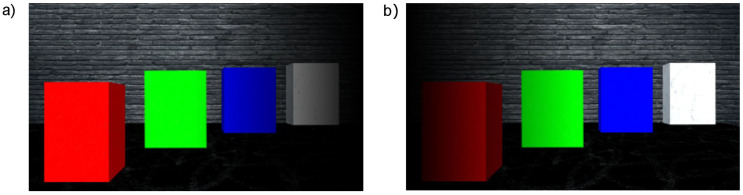
Simulated scene with applied linear functions that realise spatialised sound. (**a**) Left camera; (**b**) Right camera.

**Figure 5:**
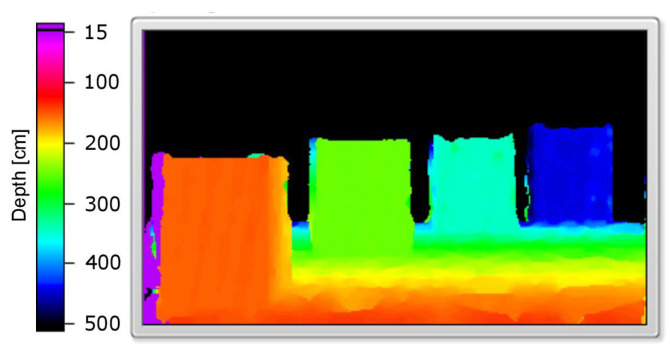
Calculated depth map.

An example of a simulated scene viewed from two cameras is presented in Figure 3. Moreover, the influence of applied linear functions for spatialising the sound shown in Figure 2 can be clearly observed in Figure 4. The scene shows four objects at a different distance to the camera, presenting basic colours: red, green and blue as well as white. The wall behind the objects is further than the depth limit, and therefore cannot be detected and shown on the depth map (Figure 5).

It was observed that, for the scenes captured by the simulated stereo system, the algorithm estimates both close and far distances accurately. Moreover, changing the parameters of the SGBM algorithm (Table 1), such as Window Size, P1 or P2 does not influence obtained results significantly. The reason might be the lack of noise or lens distortion in the simulation, whereas, in the real-life conditions, those factors are corrected by the calibration only to some extent, therefore causing some distortions in the final result.

**Figure 6:**
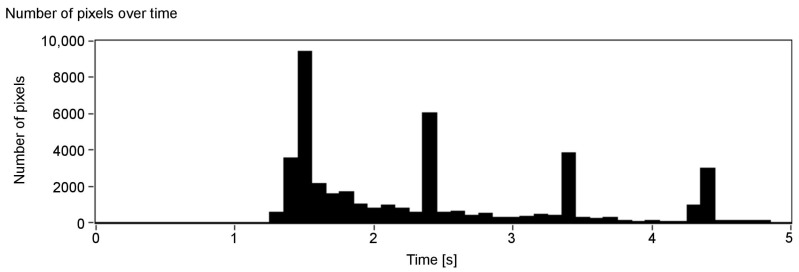
Number of detected pixels over time (each column represents one slice).

Figure 6, presenting the number of detected pixels over time, shows promising results: the peaks indicate when a surface at a given axial plane was detected. As can be seen, they can be assigned to the corresponding surfaces in the scene (see Figure 5). Absence of data for over one second at the beginning of Figure 6 is a result of the minimum depth value agreed and lack of objects—the first one is at around 150 cm (Figure 5). Note that, for the more distant objects, the number of details that can be detected is smaller (the peaks of the amplitude are less pronounced). This, however, is also true for human vision: we do not see details, especially those related to the characteristics of the spatial object, from a far distance. A possible disadvantage of the depth map calculation algorithm applied in the proposed solution is that the objects of the homogeneous colours are difficult to detect, and it might be problematic to find the corresponding points between the two images.

To allow a better understanding of the applied procedure of visual echolocation, a visualisation of the presented example was made. The scene’s axial planes are presented in the same order as they appear in the audio output link 1. Thanks to the application of the spatialised sound, there is a distinct difference depending on whether an object is presented on the left, right or in front of the user.

**Figure 7:**
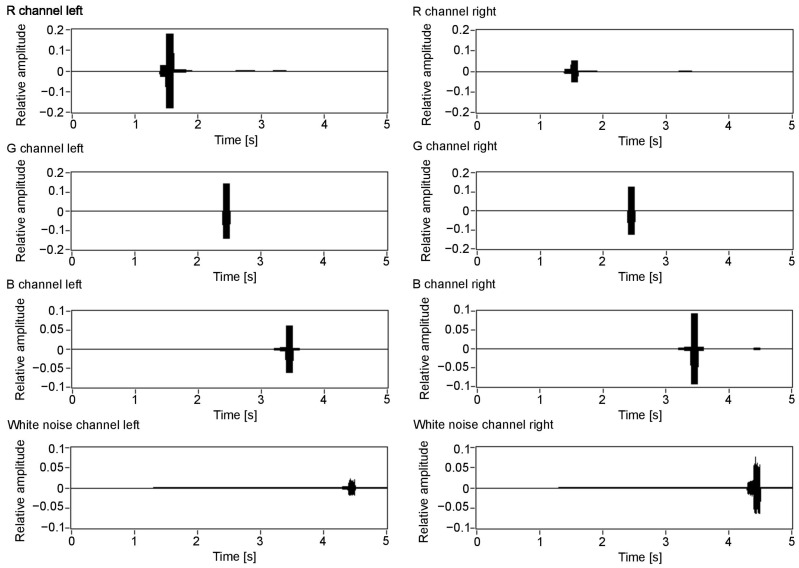
Colour value output for each of the colour components: R, G, B and white noise for the auditory channels. Value 1 in the Y scale is assigned to an object that covers the whole scene and has 255 colour intensity.

Figure 7 visualises the amplitudes of the audio output for the three colour components (R, G and B) and white noise for the left and right channels. As can be seen in Figure 3, the closest object is red, then green, blue and white. Therefore, the first detected surface is the red object. Red is a dominant colour of that part of the scene, which can be seen with the observed amplitudes: the first visible peak in both channels has the amplitude for the red component. Additionally, the observed amplitude is higher for the left channel, as this surface is visible on the left side of the scene. The increased amplitude is also observed in the right channel, although with a smaller value. This is because, for the implemented sound spatialisation, the object can be mostly heard in the left ear. Furthermore, the next peaks indicate green, blue and white, with green having the amplitudes similar for both channels, as the object meets the middle of the scene, then blue and white having the amplitudes most pronounced in the right channel, as the objects appear on the right side of the scene.

**Figure 8:**
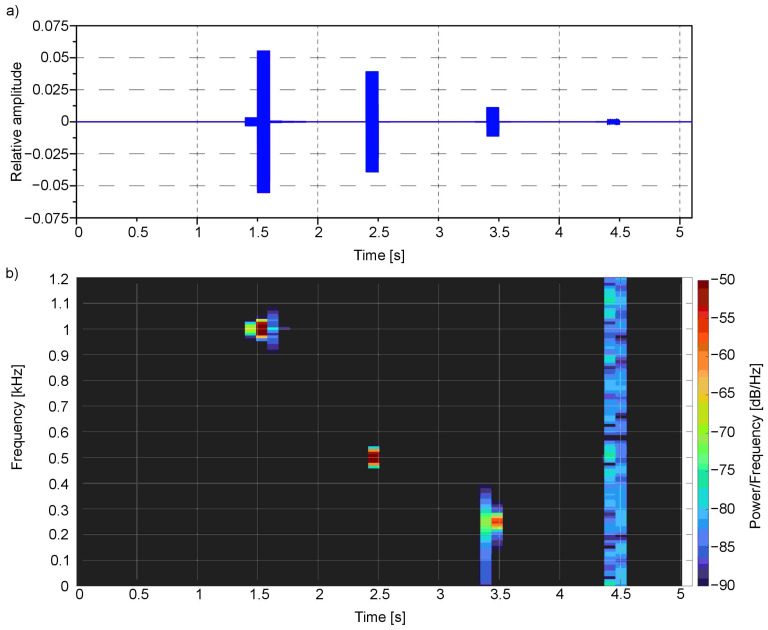
Signal from the first example (Figure 3) in (**a**) time domain and as well as (**b**) spectrogram for the left channel.

**Figure 9:**
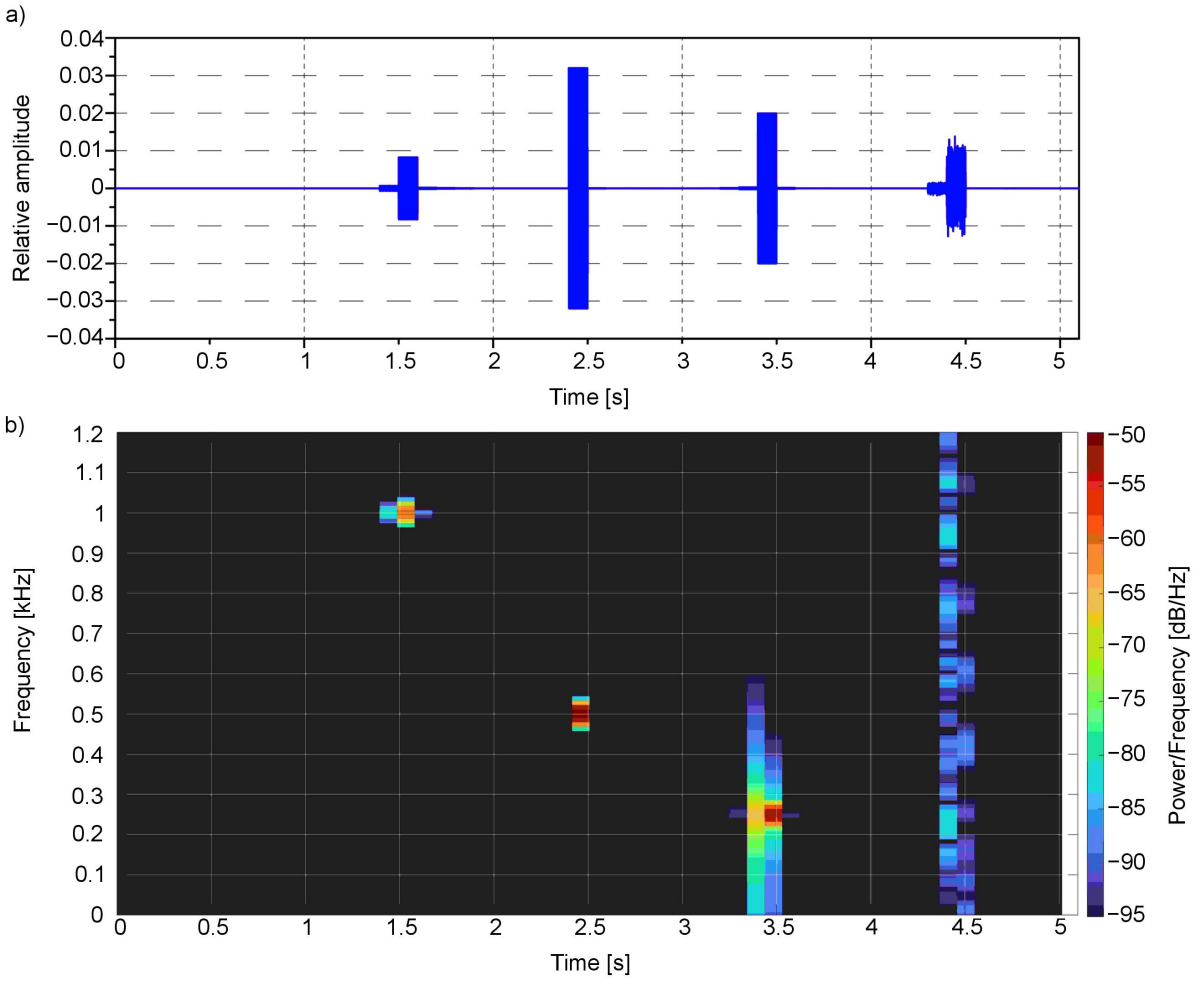
Signal from the first example (Figure 3) in (**a**) time domain and as well as (**b**) spectrogram for the right channel.

The time-frequency analysis (Figure 8 and Figure 9) was performed for the presented example. For this purpose, the Short-Time Fourier Transform (STFT) for both the left and right channels was calculated.

The spectral analysis clearly shows the main frequencies in the audio signal concerning the time of occurrence: the first one bestead around 1000 Hz in 1.5 s moment, which corresponds to red colour, the second one around 500 Hz near 2.5 s moment—for green, third one around 250 Hz near 3.5 s moment—for blue, and finally the last one—white noise that is composed of the whole spectrum of frequencies—for white colour. All these frequencies are located in a particular moment in time: 1.5, 2.4, 3.4 and 4.4 s, respectively.

**Figure 10:**
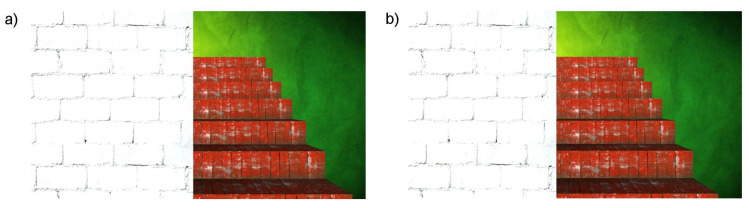
Example of the simulated simple staircase with wall. (**a**) View from left camera; (**b**) View from right camera.

Another example of a simple staircase with a wall is presented in Figure 10.

**Figure 11:**
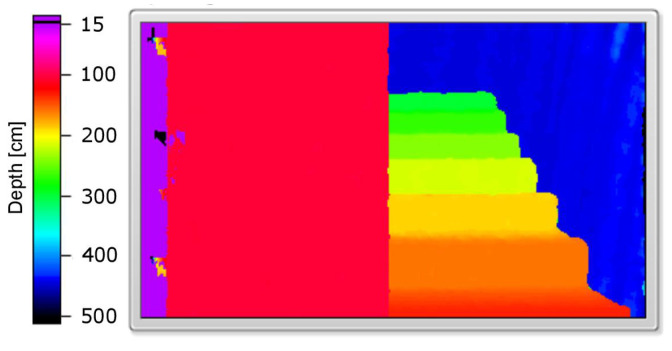
Calculated depth map.

**Figure 12:**
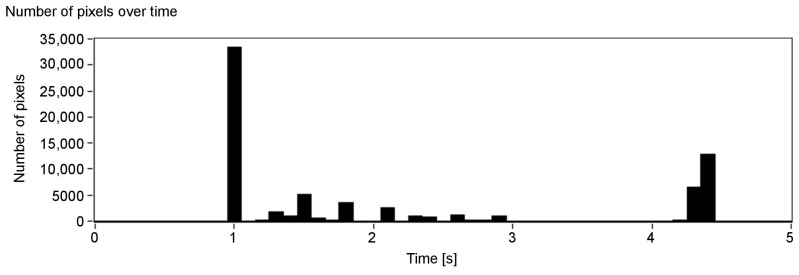
Number of detected pixels over time (each column represents one slice).

**Figure 13:**
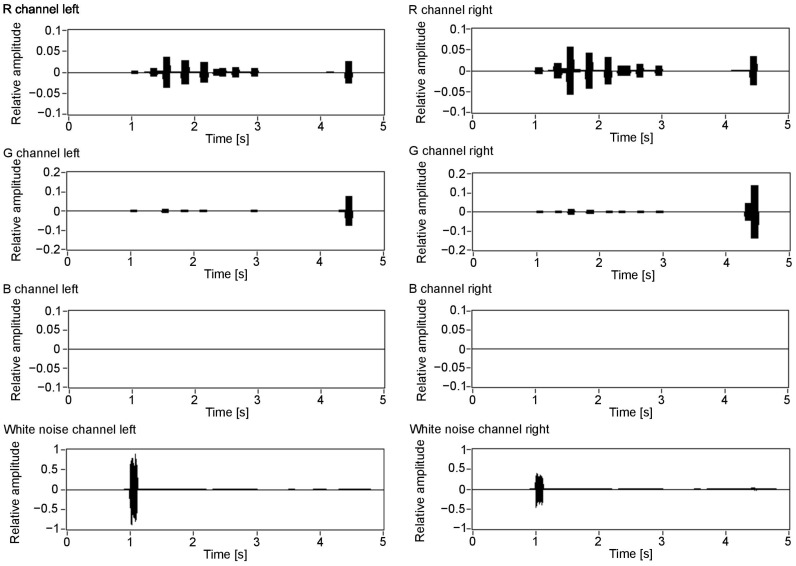
Colour value output for each of the colour components: R, G, B and white noise for the auditory channels. Value 1 in the Y scale is assigned to an object that covers the whole scene and has 255 colour intensity.

The second example (Figure 10) presents objects that can be easily recognised in the results (Figure 11, Figure 12 and Figure 13), and that is a big white wall, red stairs and a green wall. First of all, a considerable area of the front white wall is detected with the biggest amount of pixels (Figure 12). It is also noticeable in the audio output, as the appearing peak of the signal amplitude near the first second in time chart (Figure 13). The following stairs can be distinguished also according to both pixel number and audio output; moreover, green and blue canals contain almost no signal, as the dominant colour is red. Finally, in the end, the green wall can be distinguished. A visualisation of the example was presented in link 2.

**Figure 14:**
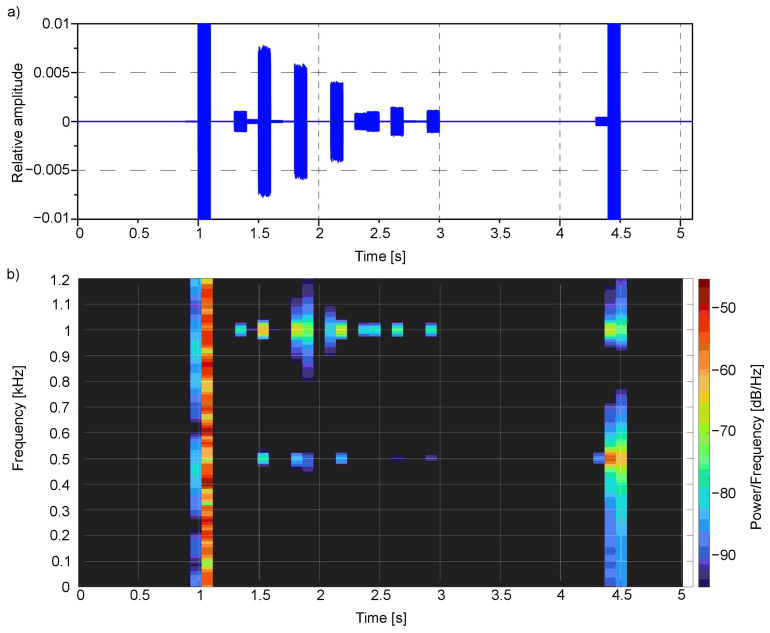
Signal from the second example (Figure 10) in (**a**) time domain and as well as (**b**) spectrogram for the left channel.

**Figure 15:**
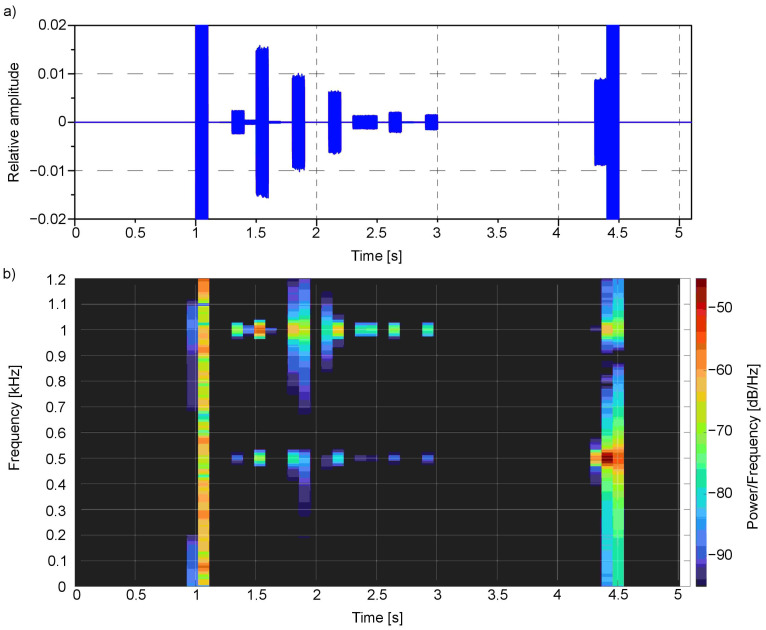
Signal from the second example (Figure 10) in (**a**) time domain and as well as (**b**) spectrogram for the right channel.

The spectral analysis for the second example is presented in Figure 14 and Figure 15.

The presented spectrograms (Figure 14b and Figure 15b) reflect the objects detected in the scene (Figure 10): first, represented by whole frequencies spectrum, the white wall that is more pronounced for the left channel as it is on the left side of the scene; then, the seven stairs whose main colour is red—1000 Hz, with a small amount of green—500 Hz and in the end the green wall with some red components. The peaks correspond to the colour values observed in Figure 13 as well as to the values observed in the time domain (Figure 14a and Figure 15a). For the presented example, the Y ordinate of the time chart was scaled in order to observe the small values of peaks. The ones around 1 and 4.5 s have higher amplitudes that exceed the scale.

To summarise, it is possible to represent the information about 2D and 3D features of a complex visual scene mimicking echolocation and using spatial auditory output. The proposed concept opens new possibilities in developing sensory substitution systems making use of scene sonification. Particularly, extending the concept of Colorophone developed by our team [3], we can develop a new tool enriching with distance information. This tool would be, to our knowledge, the first one that enables 3D features sonification of a complex scene. The disadvantage of the current version of the simulation is the computation time: now, a dozen seconds, but ideally should be much shorter. A possible solution to this could be a future implementation on a more computationally efficient platform. The approach used in this work enables the direct implementation of the developed code on hardware using field-programmable gate arrays (FPGAs).

## 5. Preliminary Tests

Preliminary tests were carried out on three participants. All of them were sighted men from 29 to 30 years old. The participants did not have any previous experience with similar audio SSDs. The tests consisted of a training part, where three scenes were presented as audio-visual stimuli with a possibility to repeat the recordings, and a test part, where three scenes were presented only as auditory stimuli also with the possibility to repeat the recordings. Videos presenting scenes used for the training part can be found under the following links: Training scene 1, Training scene 2, Training scene 3. In the test part, the difficulty level increased for every scene, starting with tasks similar to the learning tasks. Videos presenting the test scenes can be found under the following links: Test scene 1, Test scene 2, Test scene 3. The two last scenes used during the tests are the same as previously presented in this paper in Figure 3 and Figure 10. Before conducting the tests, the participants have been informed that they will have to answer the following questions about each of the test scenes:

(a) How many objects have you heard?
(b) What were the colours of the objects in the order of appearance?
(c) Which side were the objects on in the order of appearance?
(d) Draw what you have heard.

**Figure A4:**
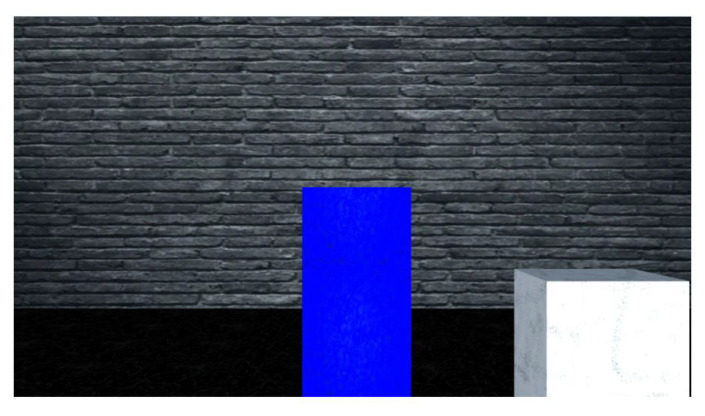
Test scene 1, view from left camera.

Detailed instructions for the preliminary tests can be found in Appendix A, while test results are summarised in Table 3. The preliminary tests showed that participants were able to interpret the scenes with minimal training. This includes identifying how many objects were presented, their colours and side that they appear. For a simple scene (Appendix A, Figure A4) consisting of two objects, all of the participants were able to detect the objects, recognising their colours and side. However, two of the participants reported that they have heard as if the first object would be separated into two. This results from the fact that the object is not a flat surface, but a 3D box, thus participants can also hear the sound generated by the sidewall of the box. For a more complex example of four objects (Figure 3) all of the participants were able to detect the number of the objects as well as their colours and sides they appear. The two objects presented in the middle (green and blue) could be interpreted as appearing on both left/right side or centre, as none of them is precisely in the middle or on the side of the scene. The most complex task presenting a semi-realistic scene with a staircase (Figure 10) had additional challenges. First of all, the number of objects that could be recognised (counting a single stair as one object) was much higher than in the previous scenes (a front wall, seven stairs and a back wall). Furthermore, because depth resolution decreases with distance and a method of a defined thickness of planes (PA parameter) was used, some surfaces may be interpreted as more than one. Additionally, sounds presenting stairs appear rapidly one after another, so it was difficult to count them. Nevertheless, the number of objects was correctly recognised (with +/−1 object accuracy). Another task given to the participants was to recognise colours. During the training, participants were presented with the primary colours: red, green, blue and white. However, in the third test scene, mixed colours were introduced. For example, the reddish colour of the stairs is composed of red and green colour components that can be observed on the corresponding spectrograms (Figure 14 and Figure 15). This could be a reason why participants misinterpret the red colour to be green. Moreover, noise added to the colours (white component) could explain why two out of three participants recognised some of the stairs or the wall at the back as white. The participants recognised the position of the objects correctly—the objects were either from left to the middle or from the middle to the right; therefore, responses “left”, “middle” and “left towards the right” or “middle/right” and “right” could be counted as correct. Finally, participants had to draw the scenes they have heard. The drawings presented in Appendix B show that the participants were able to identify objects in the scenes after a short training, recognising their order and side on which they appear (left, middle or right).

It is worth mentioning that the preliminary tests are not a base to evaluate the system, but the very concept of the visual echolocation. One of the reasons is that a prototype was tested, rather than a system based on an actual 3D camera. Therefore, the scenes were not natural, with simulated noise present in real-life observed only in the third test scene. In conclusions, the results of the tests confirmed that the prototype needs some improvements, such as implementing a more advanced head-related transfer function (in order to recognise more than the three sides of a scene) or adjusting the PA parameter in order to adjust for the thickness of the processed plane.

## 6. Conclusions and Future Work

The proposed concept of visual echolocation brings promising results. Sonification of colour and distance scene was successfully implemented and provided useful information about the complex visual scene. The concept has been evaluated on three sighted participants. All drawings and comments made by the participants are collected in Appendix B.

The results show that the proposed sonification method is easy to learn. After a short training period, participants can recognise the information about the colour and the position of the sonified objects. Some of the participants were able to perceive the sidewall of the first test object in the Test scene 1 and wondered if this represents one or two objects. Participants had no problems with analysing the Test scene 2 that was more complicated than the scenes used during the training. However, as expected, they were not able to fully guess what was presented in the Test scene 3. One of the participants suggested that the scene represents a wall of a building, but was not able to recognise that the next object was a staircase. Nonetheless, despite difficulties with guessing what is represented in the Test scene 3, drawings made by the participants almost correctly represent the sonified information. One of the probable sources of the confusion was the fact that complex colours not introduced during the training were used. Additionally, the choice of the sonification frequencies that were harmonically related might be misleading. This selection could induce the perception of the lowest harmonic component of the auditory signal being the dominant one, thus resulting in perceiving reddish/orange stairs as green. The described issue can be addressed by choosing sound frequencies that correspond to colour components in such a way that the sounds are not harmonically related. In other words, dissonant sounds should be used. It was interesting to notice that the participants were using colour cues in their strategies connected to the analysis of the third scene. Another task that was difficult to perform for the participants was the task of object counting. Although the results are satisfactory, it seems that the current setting for the moving speed of the axial plane is too high for the participants in order to enable effortless object counting when they appear rapidly one after another. The possible solution to this problem is the adjustment of the moving speed of the axial plane so the participant will have enough time to count appearing objects. In a broader context of designing the future functionalities of the system, the concept of giving the user control over the moving speed of the plane is worthy of consideration.

The disadvantages of the current solution are the inability to decouple the colour intensity from the size of the object and the possibility that equidistant objects will be interpreted as one. These misinterpretations may appear because of the superposition of the sonified information about the complex colours (e.g., it will be impossible to distinguish one violet object from two objects—blue and red ones that are equidistant to the user). These disadvantages can be compensated by using a point colour sonification provided by the Colorophone system. Moreover, the potential disadvantage of decreased resolution for further distances will discriminate against small objects that are in fact not crucial for navigation or orientation. The pronounced objects will therefore be either big or small, close to the user, and with intense colour. Another issue comes from the potential confusion created by two equidistant objects of the same colour that are placed on both the left and the right side of the processed scene, which could be perceived as one object. This issue can be addressed by the implementation of different musical instruments for the left and right channels and the implementation of a more sophisticated head-related transfer functions. It will enhance the performance of the system when it comes to object localisation and masking effects. It is also worth mentioning that the situations described above are special cases that will manifest themselves under specific conditions. The enactive, iterative use of the system by changing the viewing perspective will change the relative position of the objects and the user, resulting in the reduction of effects mentioned above.

The main advantage of the system is the possibility of sonification of the entire colourful scene in a rather intuitive way. This feature of the system distinguishes it from echolocation-inspired systems described earlier. Although the Sound of Vision system [5] and the system presented by Bujacz et al. [6] provide real-time information about the distance and position of the objects, both lack information about the colour. On the other hand, the main functional difference between our system and EyeMusic [4] is the lack of depth information in the latter one. Systems presented by Hamilton-Fletcher et al. [22] and González-Mora et al. [9] provide the distance information and a simplified representation of colour, although the latter one is either represented as black and white or grayscale. The most similar solutions proposed by Hub et al. [10] and Gomez et al. [7] sonify both distance and colour information; however, the first one provides feedback in a form of speech of the front-most object, and the latter one sonifies at most a single row of the image or a few chosen points from the scene. To conclude, other SSDs that have been introduced in the paper do not combine colour and depth information of the whole scene. It is therefore difficult to directly compare our results with results from other systems.

Because the scenes were captured in the virtual environment, where all the parameters are fully controllable, the results have high accuracy. Promising results of the simulation allow for further development of the proposed solution. The first step would be to utilise a real 3D camera and compare the precision of the results with those presented in this paper. Moreover, some parameters could be still optimised, such as plane averaging or computation time. One of the potential solutions that will reduce computational time is a further reduction in image resolution. Another possibility is related to the compatibility of the used programming platform—NI LabVIEW that can also be used for programming of real-time FPGA (Field Programmable Gate Array) platforms like CompactRIO.

## Figures and Tables

**Table 1:** Parameters used for the experiments.

		Parameter Name	Value
Camera		Resolution of a single picture	1104 × 621/368 × 207
		Field of View	H: 76${}^{\circ }$, V 47${}^{\circ }$
		Baseline	6.3 [cm]
		Aspect Ratio	76:47
SGBM Algorithm and Interpolation	General	Depth limit	500 [cm]
		Minimum depth value	15 [cm]
		Filter Cap	15
		Process Band	True
		Order	1
		Sampling Frequency	25
	Correspondence Options	Method	SAD
		Window Size	21
		Minimum Disparity	0
		Number of Disparity	160
		P1	100
		P2	500
		Full DP	False
		Max Left Right Disparity	5
		Subpixel	True
		Invalid Disparity Replace Value	−16
	Postfilter Options	Texture Threshold	15
		Uniqueness Ratio	10
		Speckle Window Size	1
		Speckle Range	500
Echolocation parameters	Main sound coding colour	Amplitude R	1
		Amplitude G	1
		Amplitude B	1
		Frequency R	1000 [Hz]
		Frequency G	500 [Hz]
		Frequency B	250 [Hz]

**Table 2:** Execution time for different image resolutions.

Image Resolution for Depth Map Calculation	Image Resolution for Further Processing	Execution Time for Depth Map Calculation [s]	Execution Time for the Entire Process of Visual Echolocation [s]
2208 × 1242	-	8.5	-
1104 × 621	-	2.3	-
736 × 414	-	1.1	-
1104 × 621	736 × 414	-	>180
1104 × 621	368 × 207	-	24
1104 × 621	184 × 104	-	8.6

**Table 3:** Preliminary tests results. Used abbreviations: R—red, G—green, B—blue, W—white, M—male.

Task/Question	Correct Answer	Participant 1	Participant 2	Participant 3
1.a)	2	2	2	3
1.b)	1-W 2-B	1-W 2-B	1-W 2-B	1,2-W 3-B
1.c)	1-right 2-middle	1-right 2-middle	1-right 2-middle	1,2-right 3-middle
2.a)	4	4	4	4
2.b)	1-R 2-G 3-B 4-W	1-R 2-G 3-B 4-W	1-R 2-G 3-B 4-W	1-R 2-G 3-B 4-W
2.c)	1-left 2-left/middle 3-right/middle 4-right	1-left 2-middle 3-right/middle 4-right	1-left 2-middle 3-right 4-right	1-left 2-left/middle 3-right/middle 4-right
3.a)	9	10	8	9
3.b)	1-W 2-8-R 9-G	1-W 2-10-G	1-W 2-6-G 7-W 8-B	1-W 2-6-G 7,8-B 9-W
3.c)	1-left/middle 2-10-right/middle	1-middle 2-10-right	1-middle 2-8-right	1-left towards right 2-9-right
**No. of trials**				
Scene 1		3	3	3
Scene 2		3	3	4
Scene 3		3	3	2
Test 1		3	5	3
Test 2		5	11	6
Test 3		8	18	8
**Age**		30	29	29
**Gender**		M	M	M

**Figure A1:**
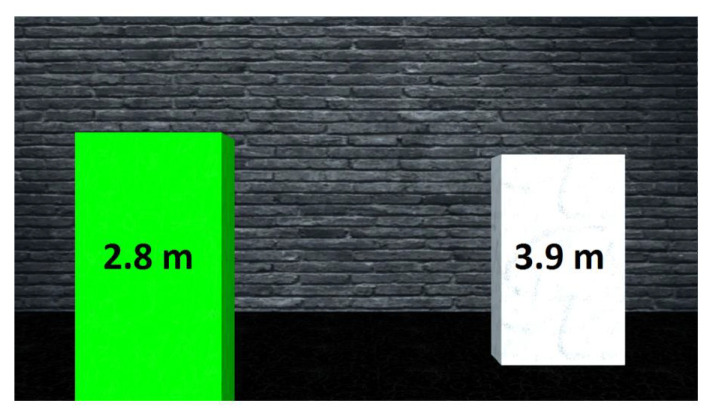
Training scene 1, view from left camera.

**Figure A2:**
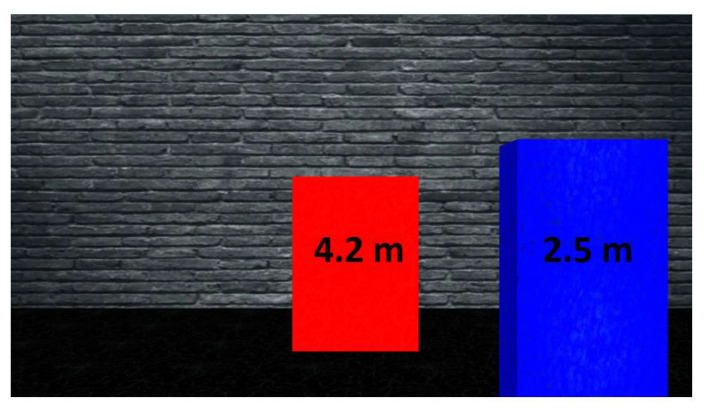
Training scene 2, view from left camera.

**Figure A3:**
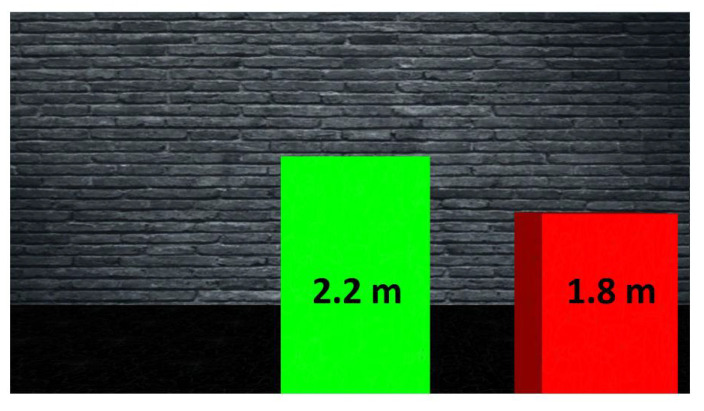
Training scene 3, view from left camera.

**Figure A5:**
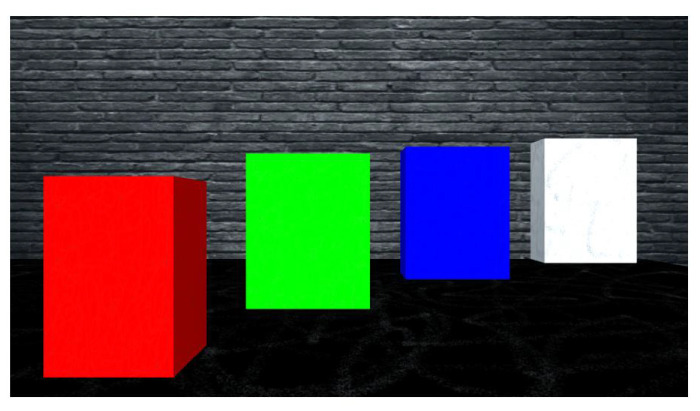
Test scene 2, view from left camera.

**Figure A6:**
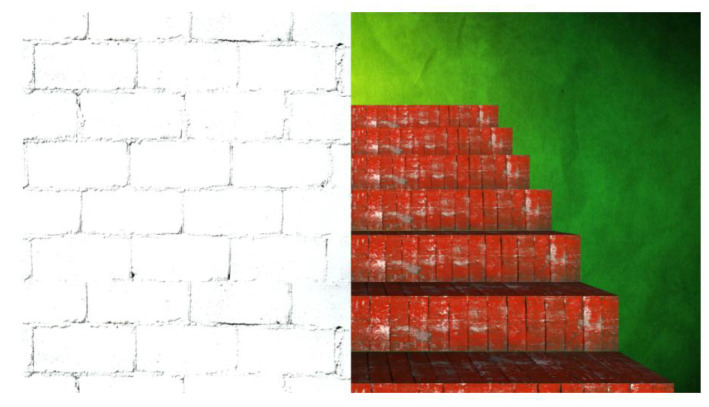
Test scene 3, view from left camera.

**Figure A7:**
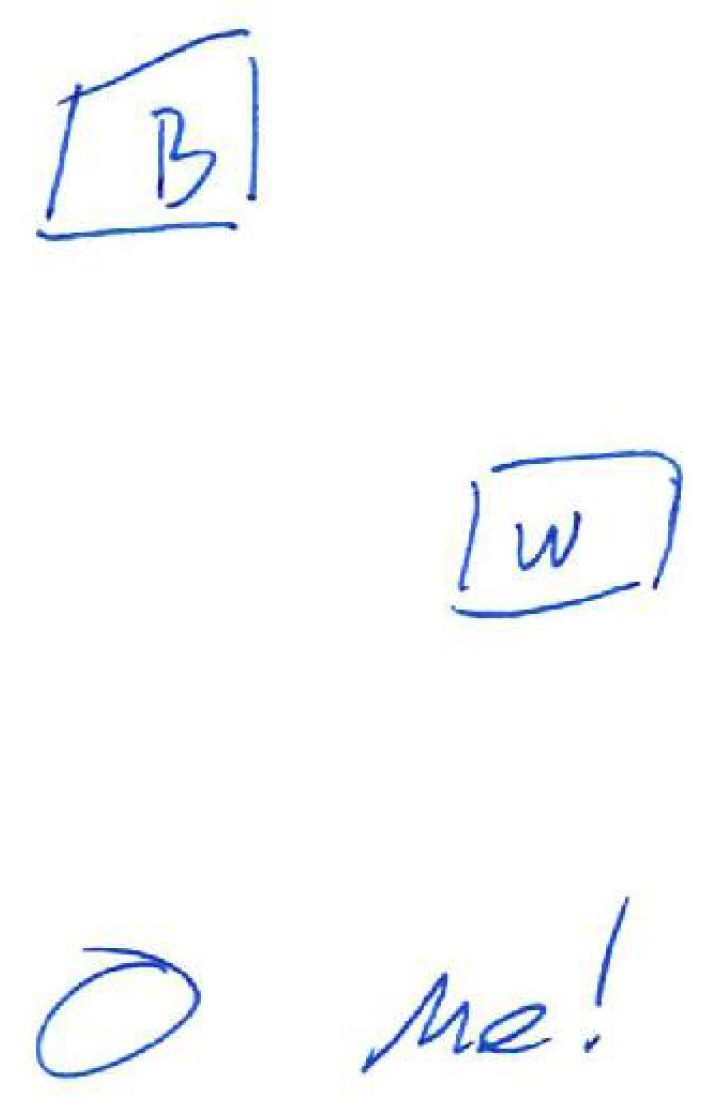
Participant 1—Test scene 1. The participant commented: “White sounds like two objects very fast after one another.”

**Figure A8:**
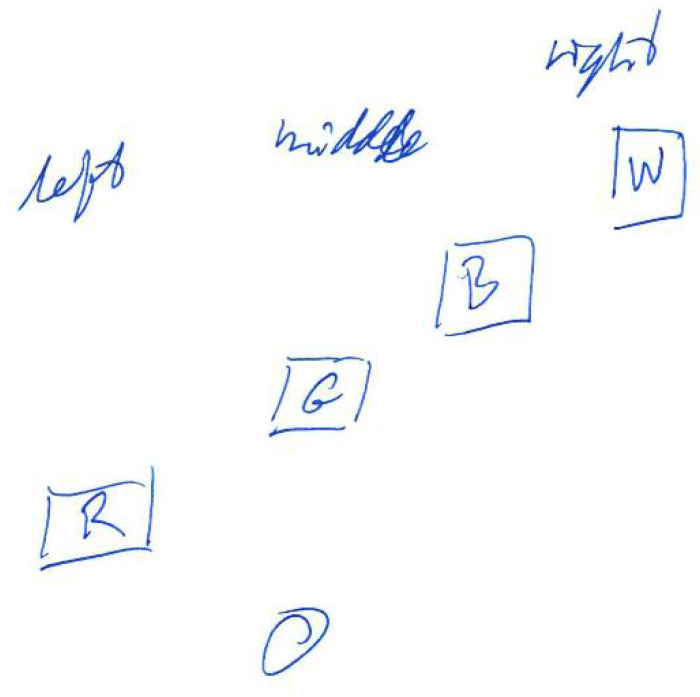
Participant 1—Test scene 2. The participant commented: “Backwards echo in red sound.”

**Figure A9:**
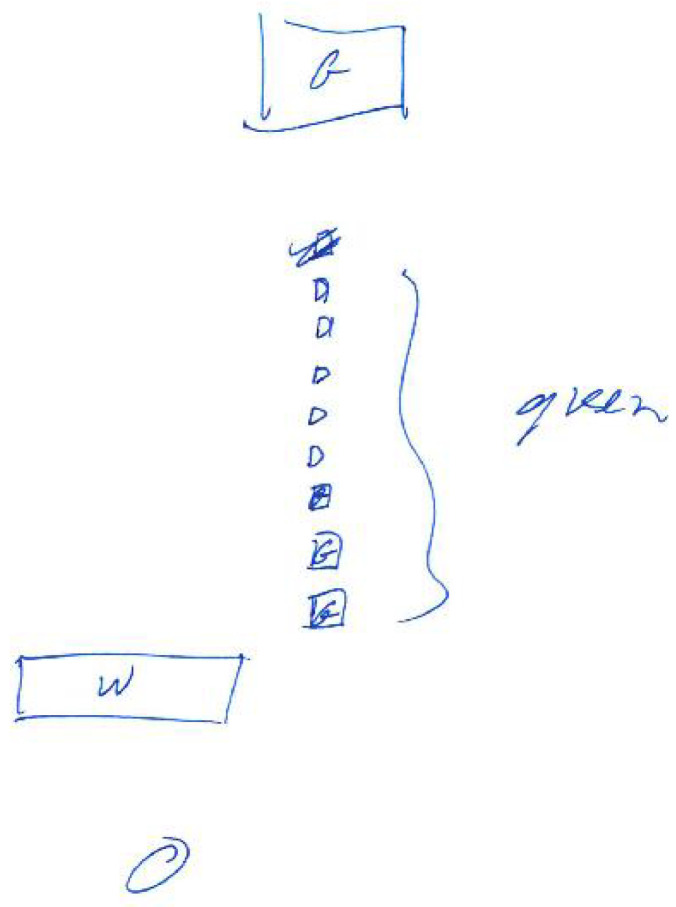
Participant 1—Test scene 3. The participant commented: “White obstacle in the middle then some small "light post" after one another then some large green object furthest away.”

**Figure A10:**
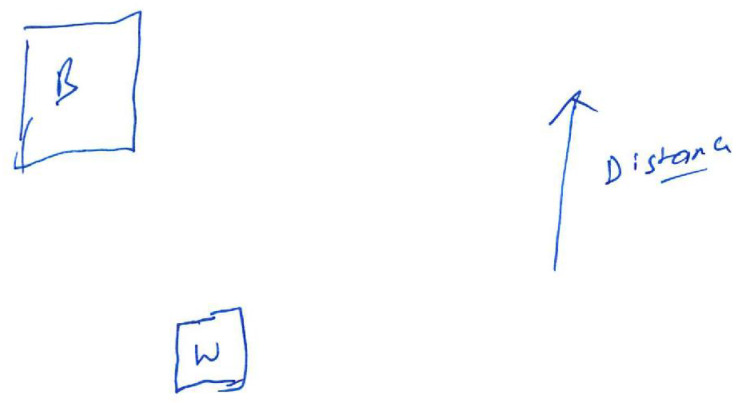
Participant 2—Test scene 1.

**Figure A11:**
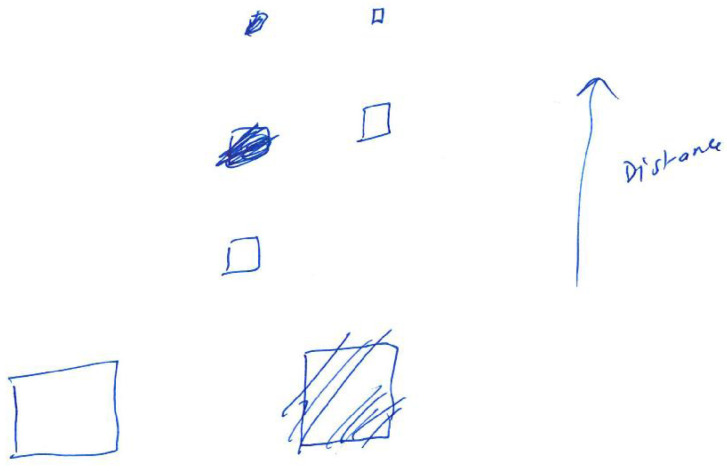
Participant 2—Test scene 2.

**Figure A12:**
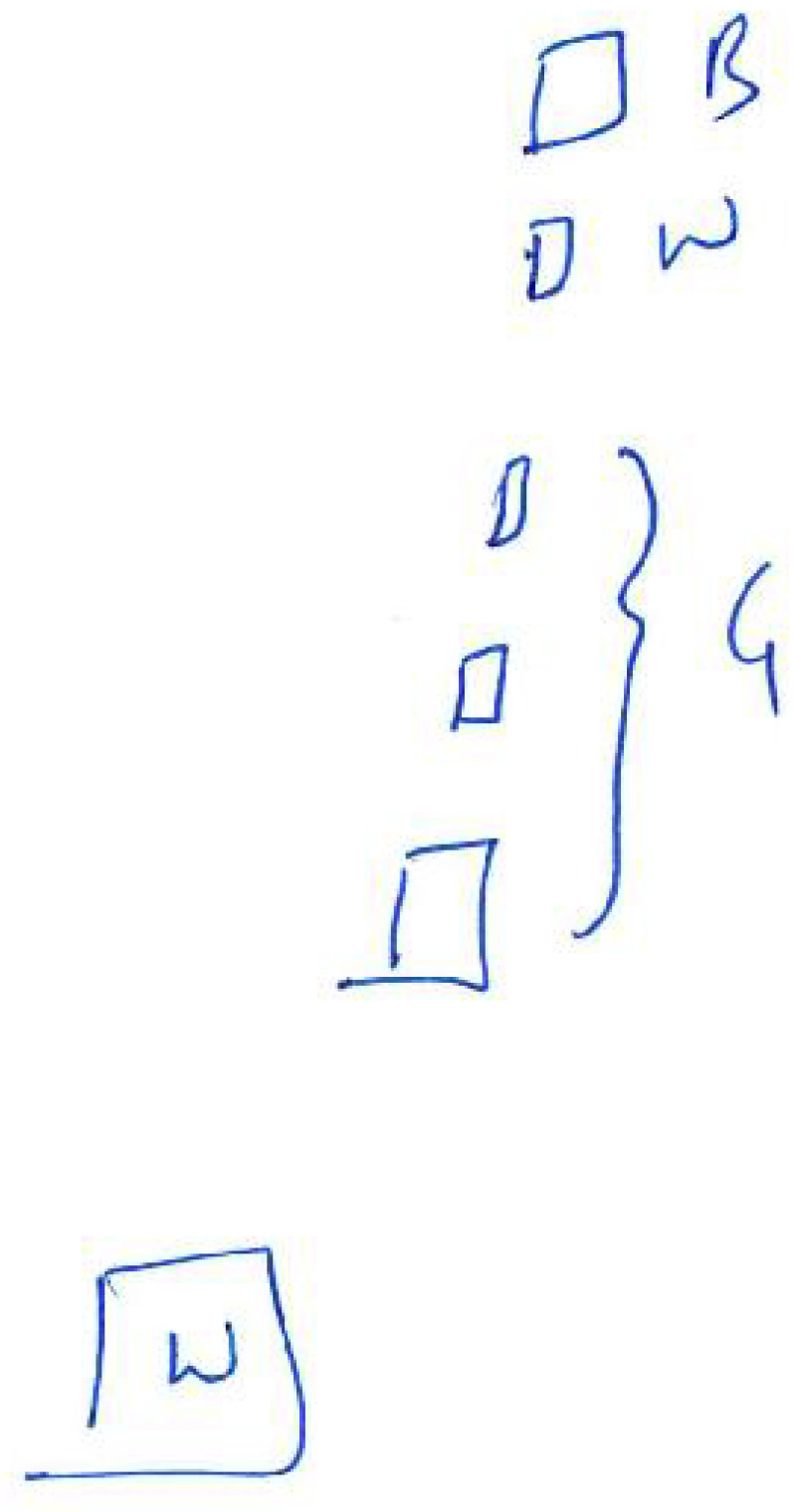
Participant 2—Test scene 3.

**Figure A13:**
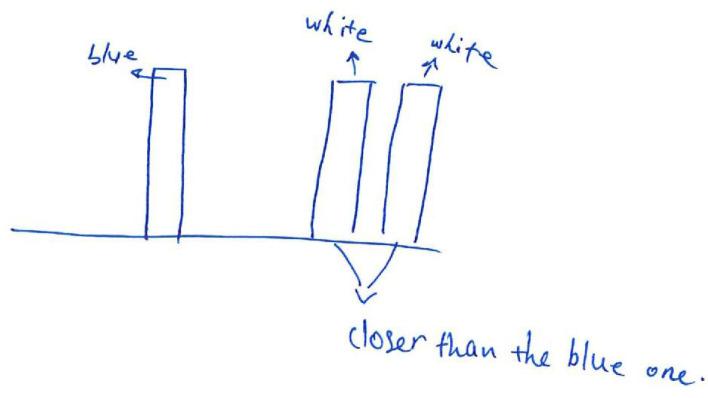
Participant 3—Test scene 1. The participant commented: “Just not sure if the first part was two different white objects.”

**Figure A14:**
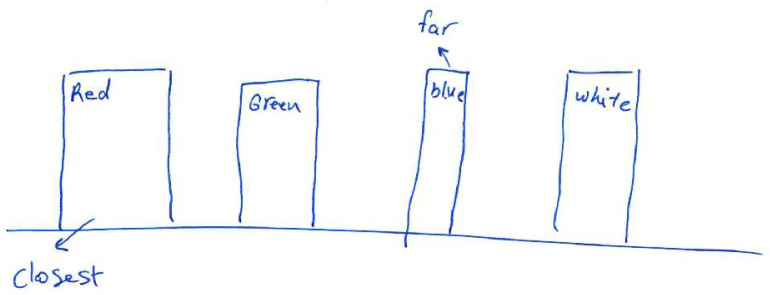
Participant 3—Test scene 2.

**Figure A15:**
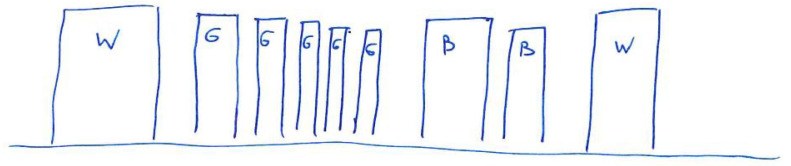
Participant 3—Test scene 3. The participant commented: “It starts with white (close) which can be some building’s wall then start green which is open air tree/bushes and a bit of blue that can a some blue car passing by.”

## Data Availability

Data sharing not applicable.

## Appendix A. Instruction for Preliminary Experiments with the Colorophone Visual Echolocation System

The purpose of the test is to evaluate the effectiveness of the proposed system. It aims to convert the information about what we see using an algorithm working similarly to echolocation. In the future, the system will be used by visually impaired people in order to extend their perception of their surroundings. The tool is based on a stereo camera that captures a scene that is then converted into an audio signal. The tool allows for auditory presentation of colour, size and distance of the objects within the scene.

First, a concept of sonification will be introduced. Then, echolocation will be explained together with some examples. Participants will use stereo headphones for listening to the stimuli.

1. Introduction to the sonification method of Colorophone
  Sonification means using sound other than speech to pass information—in our case visual information. In short, the scene that a sighted person can see, a visually impaired one can hear. To understand the presented sounds, one needs to get familiar with the sonification method of Colorophone (an assistive device for blind people). Namely, there are three basic sounds used—for red, green and blue; additionally, white is heard as white noise and black is represented by silence. The higher a given primary colour’s intensity, the louder the sound corresponding to that colour is. The rules of coding more complex colours are not needed for the test. In the tests, there will be no black objects.
  For the experimenter:
  At this moment, the sounds for red, green, blue and white are presented multiple times, without visual presentation. Next, the participant is asked to randomly recognise the colours, to make sure they can distinguish them.
2. Introduction to visual echolocation, training
  In order to understand how the tool works, it is also necessary to introduce the concept of echolocation. In nature, echolocation serves as a tool to find a location of objects in the environment. In short, first, a sound is emitted that, after reflecting from objects, returns to the ears, and, based on the perceived direction, return time and the intensity of the coming sound, it can be interpreted to obtain direction, distance and size of the objects. Such a method is used for orientation by bats or dolphins.
  In the evaluated tool, there is a stereo camera obtaining information instead of the sound emitter and receiver. The visual information is then converted to an audio signal, mimicking the concept of echolocation. One can imagine it as an axial plane moving from the user that moves through objects in the scene. Whatever object the axial plane crosses at a given moment is being sonified. The sonification is realised by using the following rules:
  - time defines distance—the later something is heard, the farther it is from the camera,
  - the area that the mentioned plane encounters is analysed by averaging the colours it contains. In other words, if it contains, for example, both red and white colours, we will hear the output sound corresponding to the appropriate amount of red and white,
  - the loudness of the sound depends both on the colour saturation (as in the sonication method mentioned before) and the size of the object (the larger the object, the louder the sound).
  Moreover, the first 15 cm starting from the camera are not sonified (silence), and the maximum distance of the sonified objects is 5 m—everything that is further is not analysed.
  Training part: Now, the test scenes will be presented with both audio and visual output. You can take notes during the training. The participant should play the recordings himself/herself in order to control the start of the sonification. Each scene shows two objects placed on the floor—while listening to the recordings, the participant should pay attention to:
  - position of the objects (more from the left side, in the middle or more from the right side),
  - distance of the objects (closer, further),
  - distance between the objects,
  - colours (red, green, blue, white).
  For the experimenter:
  At this point, Training scenes 1 (Figure A1, Training scene 1), 2 (Figure A2, Training scene 2) and 3 (Figure A3, Training scene 3) should be presented as audio-visual stimuli. There is a possibility of listening to the recordings several times. The experimenter should describe the characteristics of the scenes to the participant about the features mentioned above: which object is located where, objects are near/far, objects are closer/further to each other, what are the colours of the objects. Additionally, write down any comments on tasks. The participant should play the recordings himself/herself in order to control the start of the sonification.
3. Test part
  There are three tasks in the test part. Before starting each task, the participant gets familiarised with the questions that he/she will have to answer after each task. The tasks are to listen to the recordings but without access to the visual information. The participant may listen to the recordings several times if necessary. He/She can take notes during the test. The participant should play the recordings himself/herself in order to control the start of the sonification.
  For the experimenter:
  Provide the participant with an answer form, 3 blank A4 pages and a pen. At this point, questions for Test scene 1 (Figure A4, Test scene 1) should be presented, followed by a recording without visual information. The experimenter writes down the number of times the recording has been played. The participant should play the recordings himself/herself in order to control the start of the sonification.
  Questions 1:
  (a) How many objects have you heard?
  (b) What were the colours of the objects in the order of appearance?
  (c) Which side were the objects on in the order of appearance?
  (d) Draw what you have heard.
  For the experimenter:
  At this point, questions for Test scene 2 (Figure A5, Test scene 2) should be presented, followed by a recording without visual information. The experimenter writes down the number of times the recording has been played. The participant should play the recordings himself/herself in order to control the start of the sonification.
  Questions 2:
  (a) How many objects have you heard?
  (b) What were the colours of the objects in the order of appearance?
  (c) Which side were the objects on in the order of appearance?
  (d) Draw what you have heard.
  For the experimenter:
  At this point, questions for Test scene 3 (Figure A6, Test scene 3) should be presented, followed by a recording without visual information. The experimenter writes down the number of times the recording has been played. The participant should play the recordings himself/herself in order to control the start of the sonification.
  Questions 3:
  (a) How many objects have you heard?
  (b) What were the colours of the objects in the order of appearance?
  (c) Which side were the objects on in the order of appearance?
  (d) Draw what you have heard.
  For the experimenter:
  Ask the participant an additional question to Test scene 3: The last test scene presents a natural scene that can exist in reality. What could it be in your opinion?
